# Supplementary material for: Rapid turnover of life-cycle-related genes in the brown algae
Source: Genome Biol. 2019 Feb 14;20:35. doi: 10.1186/s13059-019-1630-6 (PMC6374913; doi:10.1186/s13059-019-1630-6)
Supplement: Supplementary file 3 — Protein alignments, corresponding Gblocks html file and CDS sequences for the lineage conserved gametophyte-biased genes. (ZIP 2109 kb) [file 13059_2019_1630_MOESM3_ESM.zip › Con_GA_alignments/Ec-01_011670.aa_ali.fasta-gb.htm]

Ec-01\_011670.aa.fasta.trans.aa\_ali.fasta


## Gblocks 0.91b Results

Processed file: **Ec-01\_011670.aa.fasta.trans.aa\_ali.fasta**  
Number of sequences: **2**  
Alignment assumed to be: **Protein**  
New number of positions: **408** (selected positions are underlined in blue)

```
                         10        20        30        40        50        60
                 =========+=========+=========+=========+=========+=========+
Ec-01_011670     MYGSTSARALDSSRGSGLAERKEQVQKEILAQKELIEKLEGRVASLSGRGGHRAAPRGSS
MSTRG.19657.1    -----------------------QVQKEILEQKALIEKLEGRVASLSGRGRRSTGTRVSS
                                        #####################################


                         70        80        90       100       110       120
                 =========+=========+=========+=========+=========+=========+
Ec-01_011670     -APPQEESERGTTG-RHDIEDRPATT------------------------TDAKQPHGE-
MSTRG.19657.1    GSPLSDDTGRGTSPRRQGTDARPATTPDLECVTVGQGNGDGEVTSNEQRSTPKERPSTEE
                   ##########   ###########                                  


                        130       140       150       160       170       180
                 =========+=========+=========+=========+=========+=========+
Ec-01_011670     --EGDHDLDGSARAKANSQSRGKGKSCCQEVADHGPNARRDSLRRSSQGESQAGYRGQQR
MSTRG.19657.1    RKEESHDLNANGGARTGNKPSFRRTSRAKEVADHGPNARRDSHHVAS---TSALHGANDR
                   ############           ######################     ########


                        190       200       210       220       230       240
                 =========+=========+=========+=========+=========+=========+
Ec-01_011670     TEQCAVEIQRNVRGASRRKRFLTLQAAREELEHHLQALVSAAATALAHRQH--QQDQQPN
MSTRG.19657.1    EELSAVSIQRNVRGIGRRKRFLTLRSAREELERHLLALVSVADAALTQQLQREQQQEQPR
                 ##############################################              


                        250       260       270       280       290       300
                 =========+=========+=========+=========+=========+=========+
Ec-01_011670     DGR---GAEVDSEGKAKNVSIAAERLLETSREDGLAKPPFEILRAMYCGLSEVTLAVECL
MSTRG.19657.1    IGAGAVGERGDSESRAMAVSAAALRLLDTSRKDGLAKPPFEILRAMYSCIDDIAAGLASY
                       #########################################             


                        310       320       330       340       350       360
                 =========+=========+=========+=========+=========+=========+
Ec-01_011670     SAAEEEEAGGVSGRGRNARISGLPCAATTAESCDAAKEALDAIREAVGADTTVIGETAHS
MSTRG.19657.1    SRPFRGDARSDSSAGRRT------WEVTLMESCDAAKEAMDAIREAVGADTEAVVECTSS
                 ################           #################################


                        370       380       390       400       410       420
                 =========+=========+=========+=========+=========+=========+
Ec-01_011670     TVQATASSPNAPRDHQYSPAGSGRAAAIAMTSARGGLLVSVDEPPTTDGESLAGKEHREV
MSTRG.19657.1    PGAAASSSPGVARDQRNSPAGSGRAAAVAMASTRGGLLVCSDDLPKSSQATLAERSSRVS
                 ############################################################


                        430       440       450       460       470       480
                 =========+=========+=========+=========+=========+=========+
Ec-01_011670     NDARNRGGSVDPGDTRPAQSVPLRSLDVERVAQLLRLHGFEEHVPGFVAQAVDGVMLSDP
MSTRG.19657.1    TESRHQNGQIYWDENRHPQSVPLRSLDVAGVARLLNTHGFEEEVPGFVSQSVDGVMLSDP
                 ############################################################


                        490       500       510       520       530       540
                 =========+=========+=========+=========+=========+=========+
Ec-01_011670     NLCEADLAELGLGGRAAEGAQDSRARMVSFFRKCQRNGVVFPVAGGSSPSFGGKVEPRTD
MSTRG.19657.1    NLCEADLAELGLGGDEADAAHDCRVRMVSLFRRSQQEGVLLPPGTESPSCFG--------
                 ####################################################        


                        550       560       570       580       590       600
                 =========+=========+=========+=========+=========+=========+
Ec-01_011670     SVHNGDSTPDDRRRNDSATLRKKEMGGLEGSVPGESKTDRTESTQDSPWRRGSVLVDREN
MSTRG.19657.1    ----------------------------------EGFGSKSNE--------------RND
                                                                             


                        610       620       630       640       650       660
                 =========+=========+=========+=========+=========+=========+
Ec-01_011670     QRVIAQISLDSSDTGRGDATAFTGTEGGRRMSVKLNQGVVITAGGREAGSVENMYELPAI
MSTRG.19657.1    ER-------------------YAG------------------------GSI---------
                                                                             


                        670       680       690       700       710       720
                 =========+=========+=========+=========+=========+=========+
Ec-01_011670     NAGEGELAASLEAAAAAAATAAVGKGQEGGDGAAAAVRGSENSPAGNRGRRTSLGLPTVT
MSTRG.19657.1    ------------------------------------------------------------
                                                                             


                        730       740       750
                 =========+=========+=========+======
Ec-01_011670     LYKESNGGLDSEEAGGMPIPPGVPVTTANSTVNVFR
MSTRG.19657.1    ------------------------------------
```

```
Parameters used
Minimum Number Of Sequences For A Conserved Position: 2
Minimum Number Of Sequences For A Flanking Position: 2
Maximum Number Of Contiguous Nonconserved Positions: 8
Minimum Length Of A Block: 10
Allowed Gap Positions: None
Use Similarity Matrices: Yes
```

```
Flank positions of the 9 selected block(s)
Flanks: [24  60]  [63  72]  [76  86]  [123  134]  [146  167]  [173  226]  [247  287]  [301  316]  [328  532]  

New number of positions in Ec-01_011670.aa.fasta.trans.aa_ali.fasta-gb:  408  (53% of the original 756 positions)
```
